# 

**Published:** 1827-04

**Authors:** 


### BIBLIOGRAPHICAL RECORD;

OR,

*Works received for Review between the 15th of December*, 1826, *and the 15th of March*, 1827.

1. A Physiological Enquiry respecting the Action of Moxa, and its Utility in Inveterate Cases of Sciatica, Lumbago, Paraplegia, Epilepsy, and some other painful, paralytic and spasmodic Diseases of the Nerves and Muscles. By WILLIAM WALLACE, M. R. I. A. &c. &c. Lecturer on Semeiology and Clinical Surgery, &c. Dublin. 8vo. pp. 148, with a Plate. 1827.

2. Scriptores Ophthalmologici Minores. Volumen Primum. Edidit JUSTUS RADIUS, Philos. Med. et Chir. Doct. in Acad. Lips. Med. P. P. E. &c. &c. Cum Tabula. Lipsiæ, Sumptibus C. H. F. Hartmanni. CIƆIƆCCCXXVI.

3. Pharmacopée Française, ou Code des Medicamens, Nouvelle Traduction du Codex Medicamentarius, sive Pharmacopœa Galica. Par F. S. RATIER, Docteur en Médecine de la Faculté de Paris; augmentée de Notes et Additions, contenant la Formule et le Mode de Préparation des Nouveaux Medicamens dont la pratique s'est enrichie jusqu'a nos jours, un grand nombre d'Analyses Chimiques, et suivie d'un Tableau des Eaux Minérales de France. Par O. HENRY, Fils. 8vo. pp. 556. Paris et Londres, chez M. Bailliere. 1827.

*We have to thank M. Bailliere for this volume.*

4. Observations on the Surgical Pathology of the Larynx and Trachea, chiefly with a view to illustrate the Affections of those Organs which may require the Operation of Bronchotomy; including remarks on Croup, Cynanche Laryngea, Foreign Bodies in the Windpipe, Wounds, &c. &c. By WILLIAM HENRY PORTER, A. M. M. R. C. S. in Ireland, &c. 8vo. pp. 283. Dublin, 1826.

*Journals received in Exchange.*

We have much pleasure in announcing the following amicable interchange of periodical labours, viz.

1. The Edinburgh Medical and Surgical Journal, (Quarterly).
2. The Philadelphia Journal of the Medical and Physical Sciences, (Quarterly).
3. The Revue Medicale et Journal de Clinique, (Monthly).
4. The Edinburgh Journal of Medical Science, No. 5, (Quarterly.)
5. The London Medical and Physical Journal, (Monthly).
6. The London Medical Repository, (Monthly).
7. The North American Medical and Surgical Journal (Quarterly).
8. The American Medical Review and Journal, (Quarterly).
9. The Archives Generales de Medicine, (Monthly).

10. The Journal Complementaire des Sciences Medicales, (Monthly).

11. The Journal Generale de Medicine, (Monthly).

12. Magendie's Journal de Physiologie, (Quarterly).

13. The American Medical Recorder, (Quarterly).

14. The New York Medical and Physical Journal, (Quarterly).

15. The Journal Universel des Sciences Medicales, (Monthly).

16. The Bibliothéque Medicale, (Monthly).

17. The Bulletin des Sciences Medicales, (Monthly).

18 The Magazine of Medical Literature and the Healing Art. By Drs. Gerson and Julius. (Two monthly).

---

23. Dissertatio Medica Inauguralis de Tetano. Per Joannem Wayte, M. D. Lynn-Regis, 1826.

☞ *Dr. Wayte has made elaborate researches for the various opinions of authors on Tetanus—has weighed them carefully — and judged of them judiciously. In an Appendix, Dr. W. has collected, in a tabular view, a large body of cases of Tetanus, from various publications, shewing the history, treatment, and termination of each case.*

---

24. An Essay on the Use of Chlorurets of Oxide of Sodium and of Lime, as powerful Disinfecting Agents, and of the Chloruret of Oxide of Sodium more especially, as a Remedy of considerable Efficacy in the Treatment of Hospital Gangrene; Phagedenic, Syphilitic, and ill-conditioned Ulcers; Mortification; and various other Diseases. Dedicated by Permission to the Right Honourable Robert Peel. By Thomas Alcock, Member of the Royal College of Surgeons in London; Member of the Medical and Chirurgical Society, &c. &c. 8vo. pp. 152, and one plate. Burgess and Hill, 1827.

☞ *See the present number of our Journal.*

---

25. Rheumatism, and some Diseases of the Heart and other Internal Organs; considered in the Gulstonian Lectures, read at the Royal College of Physicians, May, 1826. By Francis Hawkins, M.D. Fellow of St. John's College, Oxford, and of the Royal College of Physicians, &c. 8vo. pp. 144. London, 1826.

---

26. An Essay on Medical Education. By William Barrett Marshall, an Assistant Surgeon in the Royal Navy. 12mo. pp. 114. London, 1827.

---

27. The Medical Student, No. 1. Burgess and Hill, London, January, 1827.

---

28. Illustrations of the Arteries connected with Aneurism and Surgical Operations. These plates are intended to explain the relative position of the Arteries, in respect to the surrounding parts, and the Organs to be met with in such operations, both externally and internally to their sheaths. No. 1, containing three plates of the Anatomy of the Neck. Second Edition. No. 3, containing Plate V. Supplementary to No. 2, of the Axilla; Plate VI. Dissections 1, 2, 3, 4, and Plate VII. Dissections 1, 2. Comprising the Anatomy of the superior extremity, bend of the fore Arm and Hand. By G. D. Dermott, M.R.C.S. and Lecturer on Anatomy. London, 1827. Price 15s. each part.

☞ *These Plates are highly creditable to Mr. Dermott. The surgical anatomy of the neck and upper extremity, and the relative position of arteries, veins, and nerves to each other, are represented with clearness and accuracy. We think that the work will prove highly useful to the young surgeon, and to him we freely recommend it.*

---

29. Outlines of Human Physiology. By Herbert Mayo, Surgeon, and Lecturer on Anatomy. 8vo. pp. 406. London, 1827. Price 14s.

---

30. An Introductory Lecture to a Course of Surgery, delivered at the Richmond School of Medicine, Dublin, on the 8th day of January, 1827. By R. Carmichael, Esq. M.R.I.A. &c. Printed at the request of the Pupils. 8vo. stitched, pp. 51. London, Longman's, 1827.

31. Practical Directions for Preserving the Teeth, with an Account of the most Modern and Improved Methods of supplying their Loss; and a Notice of an improved Artificial Palate, invented by the Author. Second Edition. By ANDREW CLARK, Dentist. 8vo. pp. 96, with 6 plates. London, 1826.

---

32. Principles of Medical Science and Practice, Part II. Vol. II. Pathology. By HARDWICKE SHUTE, M.D. &c. &c. 8vo. pp. 604. London, Longman's; and Gloucester, 1826. ☞ *In our next.*

---

33. Myology, illustrated by Plates on a peculiar construction. In Four Parts. Part the Fourth. Muscles on the Face and Eye, anterior and posterior Parts of the Neck, with the Muscles of the Perineum, completing the whole of the Muscles of the Human Body. By E. W. TUSON, Lecturer on Anatomy and Physiology, Member of the Royal College of Surgeons in London, and late House Surgeon to the Middlesex Hospital. London, Callow and Wilson, 1826. Price 12s. plain, 18s. coloured, each part; or the four parts in boards, 2l. 8s. plain, or 3l. 12s. coloured.

---

34. Traité, Medico-Chirurgicale de l'Inflammation. Par S. THOMSON, Pro-Professeur de Chirurgie a Edinburgh, &c. Traduit de l'Anglais, avec des Notes, par F. G. Boisseau et A. S. L. Jourdan. 8vo. pp. 678. Paris, 1827. Imported and sold by Balliere, Medical Bookseller, Bedford-street, Bedford-square, London.

☞ *As Dr. Thomson's work is not now to be procured, and as this translation is enriched by a series of very valuable notes, we recommend it in the strongest manner to our professional brethren.*

---

35. A Lecture upon the ZOPURON, as lately delivered at the Sunderland Infirmary. By WILLIAM REID CLANNY, M.D. F.R.S.E. Physician to the Sunderland Infirmary, &c. Quarto, pp. 16. 1826.

☞ *The Zopuron (Reviver) is an ingenious apparatus, invented by Dr. Clanny, for more effectually producing artificial respiration, in suspended animation, than the bellows of the Humane Society. The lecture contains many judicious and ingenious observations on the subject of suspended animation, and the means of preventing the final extinction of the vital spark.*

---

36. Medical Botany; or Illustrations and Descriptions of the Medicinal Plants of the London, Edinburgh, and Dublin Pharmacopœias, with those lately introduced into Medical Practice; comprising their generic and specific characters, &c. Including also a popular and scientific description of Poisonous Plants, particularly those that are indigenous to Great Britain and Ireland; with Figures coloured from Nature: the whole forming a complete System of Vegetable Toxicology and Materia Medica. By JOHN STEPHENSON, M. D. and JAMES MORSS CHURCHILL, Esq. Surgeon. Nos. I. II. III. for January, February, March; to be continued Monthly, price 3s. 6d. London, 1827. ☞ *In our next.*

---

37. Memoirs sur le traitement de la Cataracte. Par LOUIS FRANCOIS GONDRET, M. D. Deuxieme Edition. Paris, 1826.

☞ *We return Dr. Gondret our thanks for this little work, and beg to refer him to our tenth Number of this series for a very full analysis of the first edition.*

---

38. Practical Observations on the Application of Lunar Caustic to Strictures of the Urethra; illustrated by Cases, proving the permanency of the Cure by that Treatment. To which are added, some Remarks on its use in Strictures in the Œsophagus. By M. W. ANDREWS, Member of the Royal College of Surgeons, London. Second Edition. 8vo. pp. 249, two plates. London, 1827. Price 9s.

---

39. Observations on the Expediency of Instituting a Friendly Association of the Medical Profession throughout Scotland, for insuring a provision during sickness and old age, &c. &c. By EDWARD D. ALLISON, Surgeon.

☞ *We are happy to see this very excellent institution carried into comple operation, for which Mr. Allison is entitled to great praise. We wish that a similar institution were formed in England; it would prevent many of those scenes of penury and distress among our brethren*

*and their desolate families, which we are daily compelled to witness. Mr. A.'s pamphlet contains all necessary information on this important subject.*

---

40. An Alphabetical Catalogue of Drugs; with a specification of their Doses to Children and Adults—the Diseases for the cure or palliation of which they are administered, &c. To which are added, a Collection of the most approved Medical Prescriptions, &c. &c. 12th Edition. By Reece and Co. London, 1827.

☞ *In this edition there is a fair account of the new chemical preparations; and the "Alphabetical List of Diseases, with reference to Remedies," is better adapted to the exigencies of the general reader (for whom it is of course designed), than most works of a popular character.*

---

41. Conversations on Anatomy, Physiology, and Surgery. By Archibald Robertson, M. D. Author of the Colloquia de Morbis, &c. &c. Highley. 18mo.

---

42. New York Medical and Physical Journal, Nos. 19 and 20, for October, 1826, and January, 1827. *In exchange.*

---

43. New York Monthly Chronicle, Nos. I. to XII. inclusive. *In exchange.*

44. A Description of the Mode of using the Forceps invented by Mr. Fay, for the extraction and excision of Teeth. 8vo. pp. 16, with Plates. 1827.

---

45. Nugæ Canoræ; or Epitaphean Mementos (in Stone-Cutter's Verse) of the Medici Family of Modern Times. By Unus Quorum. 8vo. pp. 70. Callow and Wilson. 1827.

☞ *A good humourous medley of real traits of character, and satirical glances at the foibles of the* Medici Family. *It is a twin-brother, not by the same father (if such an Hibernicism be allowed), of the* Gold-headed Cane. *It is not improbable that we shall amuse a dull hour, in our next Number, with these curious productions of the medical press.*

---

46. Elements of Chemistry, including the Recent Discoveries and Doctrines of the Science. By Edward Turner, M.D. F.R.S.E. Fellow of the Royal College of Physicians, and Lecturer on Chemistry, Edinburgh, &c. &c. 8vo. pp. 723, plates 11. Edinburgh, 1827.

☞ *This is really an excellent manual, embracing all the modern discoveries, and concentrating in a small space a vast body of chemical science, and a clear view of chemical operations.*

---

### PRIZE HOSPITAL REPORTS.

### LIST
OF
### SUCCESSFUL CANDIDATES
FOR
### THE HOSPITAL REPORT PRIZE.

Palmam qui meruit ferat.

| NO. | NAME. | HOSPITAL. | DATE. |
|---|---|---|---|
| 1 | Thos. H. Smith, (Pupil.) | St. Thomas's. | April, 1827. |

Prize—*a complete Set of the* Medico-Chirurgical Review—*free Subscription for Two Years afterwards—perpetual Registry of the successful Candidate's Name in the Journal.*

### ADDITIONAL SUBSCRIBERS.

Bartholmew, Mr. G. D. Surgeon, Reading.
Bunt, Mr. George, Carlton Chambers, Regent-street.
Curtis, Mr.
Gibson, P. C. Esq. Surgeon, Leith-walk, Edinburgh.
Giraud, S. T. Esq. Surgeon, Faversham.
Gorman, Dr. Member of the Royal College of Surgeons, Lisbon.
Haslam, Mr. T. B. Member of the Royal College of Surgeons, Carnarvon, Wales.
Henry, Mr. James, Surgeon, to India.
Hopkins, Dr. Lecturer on Midwifery, Queen-square, St. James's Park.
Irvine, Mr. John, Morville, County Donegal.
Jarman, Mr. J. Surgeon, Nottingham.
Kinnier, Dr. 7, East-road, City-road.
Morson, Mr. Surgeon, Southampton-row, Russel-square.
Muggleston, Mr. Surgeon, Grafton-street, Fitzroy-square.
"Norwich and Norfolk United Medical Book Society."—Four copies quarterly.
O'Connor, Dr. J. L. Trinidad.
Pilcher, George, Esq. Surgeon, Dean-street, Borough; Lecturer on Anatomy at Mr. Grainger's Theatre, &c. Borough.
Price, Mr. David, Surg. Margate, Corresponding Member of the London Medical and Hunterian Societies.
Sedgwick, Mr. George, Student, Borough Hospital.
Smith, Mr. John, Surgeon, R. N. Forres, N. B.
Stevens, Mr. J. Surgeon, Barford, near Nottingham.
Swineard, Mr. Frederick, Surgeon, York.
Wayte, Dr. John, Lynn-Regis.
Wilton, J. W. Esq. Gloucester.

### INDEX.

A.

Abdominal injury, fatal case of .. 472
Abernethy, Mr. his optics all in the wrong .................. 256
Abernethy, Mr. on spinal diseases 263
Abscess of the liver, M. Louis on 201
Academy of minute anatomy.... 630
Ager, Dr. his circular .......... 283
Ainslie, Dr. his materia indica .. 397
Alcock, Mr. on the chlorurets of soda and lime .............. 349
Aliments, various, considered .. 3
Alison, Dr. his observations on Sympathy .................. 63
Anatomical discovery .......... 270
Ancle, compound dislocation of the ...................... 262
Andral, M. on chronic gastritis.. 145
Amaurosis cured by long vomiting 568
Aneurism from bleeding, some cases of .................... 193
Aneurism from venesection .... 547
Angina pectoris, supposed case of 533
Aorta, obliteration of the ...... 481
Appendix to the papers on the nerves, by Mr. Bell .......... 412
Army Medical Board, regulations of the.................... 291
Ascites with pregnancy, case of.. 302
Axillary aneurism, Mr. Liston's case of .................... 579

B.

Ballingall, Dr. his case of lithotomy ...................... 83
Bals. copaibæ, extract of, in gonorrhœa.................. 499
Bampfield, Mr. his death...... 637
Barry, Dr. his reclamation...... 293
Barry, Dr. answer to .......... 620
Bell, Mr. his appendix to his papers on the nerves .......... 412
Belladonna, Mr. Chevalier on.... 231
Bile, Mr. Mayo's experiments on the ...................... 247
Bile, alterations in its state .... 515
Bische, a malignant dysentery of Trinidad ................ 470
Bitter almonds, case of poisoning by ...................... 298
Black vomit, its nature considered .................... 155
Blood, can it be the seat of disease? .................... 218
Brain diseased, without any symptoms .................... 234
Brain, state of, in hooping cough 612
Bronchia, cherry-stone in the .. 228
Bronchocele, its etiology ...... 243
Bronchotomy, Mr. Porter on.... 375
Bronchotomy in croup considered .................... 383
Broussais, M. his principles of medicine.................. 313
Broussais and Clutterbuck in danger! .................. 244
Brown, Dr. his treatment of intermittents.................. 229
Burns, Mr. Travers's treatment of ...................... 111

C.

Cancer of the penis, removal of the part.................. 551
Candidates for prize-hospital reports .................... 635
Carbonate of iron in neuralgiæ .. 500
Carditis, Dr. Gardner's case of .. 79
Caries of the teeth, Mr. Koecker's opinions on ............ 137
Carotid tied by Mr. Travers .... 165
Carotid tied *originally* by Mr. Fleming.................. 167
Caustic in inflamed absorbents .. 246
Cellular inflammation, Mr. Earle on ...................... 535
Cervix femoris, fracture of the .. 299
Cervix femoris, fracture of the .. 272
Chalmeil, Dr. on the paralysis attendant on insanity ........ 362
Chambers, Dr. his cases of fever 161
Chirurgical knowledge ........ 638
Chlorates of soda and lime, Mr. Scott on the .............. 349
Chloruret of soda, formula for its preparation .............. 350

Chlorurets of soda, and lime, Mr. Alcock on the ............... 349
Chorea, Dr. M'Andrew on ....... 189
Chronic indigestion, progress and symptoms of................. 35
Chronic gastritis, M. Andral on.. 145
Clinical report of M. Lisfranc.... 189
Clinical observations by Dr. Bland 238
Clinical medicine, Dr. Clarke's pamphlet on ................ 580
Clinique of the Hôtel Dieu...... 531
College of Surgeons, new regulations of the ................ 268
Compression in poisoned wounds 232
Concussion of the brain and nervous system ................ 105
Condiments considered ........ 8
Constitutional irritation, Mr. Travers on ................... 101
Constitutional irritation from injuries ..................... 115
Constitutional irritation from inflammation ............... 117
Constitutional irritation from loss of blood.................... 120
Constitutional irritation from animal poison................. 121
Cookery, its effects on different aliments.................... 6
Cranial fracture, bold depletion in 566
Croup, Mr. Mackenzie on the pathology of ................... 289
Cynanche trachealis, observations on ......................... 380
Cynanche trachealis, blood-letting in ..................... 382
Cynanche trachealis, bronchotomy in ...................... 383

D.

Dance, M. on intus-susception .. 176
Descot, Dr. on local affections of the nerves ................. 434
Deuto-Iodure of mercury in cutaneous affections .......... 566
Diabetes mellitus, animal diet in 238
Diagnosis and prognosis, M. Rostan on ..................... 51
Diet, Dr. Paris's treatise on .... 1
Diet of phthisical patients ...... 45
Digestion, some curious experiments on ................ 510
Disease and disorder—function and organ ................... 52
Diseases of the teeth considered 132
Disease of the heart, very instructive case of ................ 215
Dislocation of the cervical vertebræ, supposed case of ........ 613
Disorganization of the stomach; Dr. Gairdner's case .. ........ 86
Dissections, advantage of chloruret of lime in................. 354
Dissection wound, Mr. Shaw's case of ..................... 546
Drinks, different kinds of ...... 9
Dufau, M. on intermittent irritations ..................... 457
Duodenal indigestion, remarks on 31
Dupuytren, M. his case of ligature of the subclavian............ 163
Dupuytren, M. on congenital dislocation of the hip-joint...... 544
Dysmenorrhœa, Dr. Campbell on 494
Dysphagia, some curious cases of 236
Dyspnœa, chronic case of ...... 254

E.

Earle, Mr. his case of local irritation...................... 199
Edinburgh Med. Chir. Society, transactions of .............. 61
Elliotson, Dr. on the subcarbonate of iron ................ 345
Empyema, Dr. Pitcairn's case of 78
Empyema, case of ............ 255
Encephalic phlebitis, case of .... 230
Encephalitis, symptoms and pathology of ................. 409
Epilepsy, nitrate of silver in .... 241
Epilepsy, sympathetic, two cases of 245
Epilepsy, cases of, by Mr. Scott and Mr. Gunn .............. 508
Ergot of rye, effects of.......... 242
Ergot of rye in uterine hæmorrhage ...................... 257
Erysipelas œdematodes, remarks on ...................... 536
Exhumation and fumigation .... 268
Experiments on post mortem changes .................... 474
Extraction of diseased teeth and roots ...................... 214
Extra-uterine fœtus, Mr. Mackie's case ................. 496
Eye, peculiarly destructive inflammation of the .......... 342

F.

Femoral hernia, curious case of.. 611
Fever, Dr. Chambers' cases of .. 161
Fever, Dr. Hewett on .......... 198
Fever, M. Broussais' views of .. 336
Fish not so digestive as is imagined ...................... 16
Fistula in perineo cured, Mr. White's case ................ 245

Fleming, Mr. his ligature of the carotid .................. 167
Florence, a medical glance at.... 96
Fracture of the spine, case of.... 605
Fractures of the spine not *necessarily* fatal.................. 107
Fruits in dyspepsia ............ 17
Fungus hæmatodes of the left lung ...................... 519

G.

Gairdner, Dr. his case of carditis 79
Gairdner, Dr. his case of disorganized stomach ............ 86
Ganglionic and cerebral system of nerves considered............. 316
Gastritis, chronic case of ...... 261
Gastro-Enteritis, M. Broussais' view of .................. 327
Gastrotomy, a successful case of, at Parma ................. 100
Georget, M. on insanity ........ 482
Glasgow; regulations of this University .................... 294
Granville, Dr. on disinfecting liquids ...................... 629
Gregory, Dr. on vaccination .... 192
Gregory, Dr. his case of hydrophobia .................... 347
Gun-shot wound, severe case of.. 251

H.

Hall, Dr. M. on a destructive inflammation of the eye, &c..... 342
Hamilton, Dr. on a peculiar sore throat .................... 223
Hastings, Dr. on mollescence of the lungs .................. 517
Headache, two kinds of ........ 32
Heart, diseased; an important case of .................... 48
Heart, an interesting case of disease of .................... 215
Heart, Dr. Johnson's case of disease of the................. 343
Hepatitis, diagnostic symptoms of 209
Hernia, cases of, at St. Thomas's, 609
Hewett, Dr. on the treatment of fever ...................... 198
Hip-joint, congenital dislocation of ...................... 544
Hohenloë in Panton-square! .... 253
Homicide and infanticide, propensity to .................. 226
Homicide; case of Harriette Cornier...................... 482
Homicide and matricide, cases of 489
Hooping-cough, dissection...... 612
Hooping cough, case of ........ 613
Humerus, excision of its head .. 235
Hunterian Museum, Dr. Ager's circular ...................... 283
Hunterian museum, Dr. M'Cleod's circular ...................... 285
Hunterian museum, remarks on the regulations of admission to it ...................... 288
Hydrocephalus chronicus, Mr. Miller's case ................ 81
Hydrometra and dry gangrene, Dr. Thomson's case of........ 340
Hyosciamus and camphor, mode of exhibiting................ 227
Hyosciamus, effects of an overdose of .................... 242
Hypochondriasis, marked case of 466
Hysteric affections of the larynx 419

I. J.

Ileus, from calculous concretions 574
Incontinence of urine, M. Lair's remarks on ................ 244
*Independence* of organs considered 102
Indigestion, definition and symptoms of .................. 27
Induration of the cellular membrane ...................... 565
Inflamed absorbents, caustic for 246
Inflammation of the blood-vessels, cases of ................ 501
Inguinal hernia, strangulated, case of....................... 609
Injuries of the head, M. Paillard on ....................... 169
Injuries of the head, cases, with remarks .................. 263
Injuries of the head, cases of.... 529
Injuries of the head, further cases of ....................... 541
Instinct, as compared with intellect....................... 318
Intermittents, new mode of treating ...................... 229
Intermittent irritations, M. Dufau, on .................... 457
Intermittent arachnitis, supposed cases of ................ 460
Intermittent fever, cases of...... 606
Intermittent fever, modes of cure, 606
Intermittents; tartar emetic in.. 531
Internal strangulation.......... 272
Intestines, M. Paillard on wounds of the .................... 196
Intestinal ulceration after fever, Dr. Johnson's case of ........ 497
Irritability and irritation ...... 104
Irritation, Mr. Earle's case of .. 199
Iron blisters .................. 269

Intus-susception, M. Dance's memoir on .................... 176
Intus-susception, *supposed* case of 177
Intus-susception, diagnosis and treatment of................ 185
Italy, new medical doctrine ..... 626
Jowett, Mr. taps the pericardium 623

K.

Koecker, Mr. his dental surgery 129

L.

*Lancet* report; a good specimen 195
Lancet morality, or, "The Youths of Fifteen," a *farce* .......... 268
Laryngitis œdematosa, its seat and symptoms .............. 390
Laryngitis œdematosa, treatment of ........................ 393
Lawrence and radical reform; universal equality............ 267
Lawrence's lecture on the division of labour .................. 569
Lepra, venereal................ 606
Lewis Cornaro and Mr. Abernethy's golden rules............ 22
Lime, chloruret of, mode of using it as an anti-putrescent ...... 352
Lisfranc, M. his extirpation of the parotid .................. 564
Lithotomy, high operation in; Dr. Ballingall's case ............ 83
Lithotomy, Mr. Lizars' observations on.................. 273
Lithotomy, some cases of . .... 549
Local affections of the nerves.... 424
Louis, M. on abscess of the liver 201

M.

M'Cleod, Dr. his circular ...... 285
Mackenzie, Mr. on opthalmia .. 173
Mackenzie, Mr. on the pathology of croup...................... 289
Manual of pathology, M. Martinet's ...................... 407
Materia indica, by Dr. Ainslie .. 397
Maxilla inferior; partial excision of ........................ 258
Meals, hours of............... 20
Medical literature of the Hindoos 405
Medical examinations and education....................... 583
Medical periodical press, retrospections of the.............. 300
Medicine, state of, at Naples.... 92
Medicine and surgery, division between..................... 569
Medico-Chirurgical Transactions 340
Menstruation, vicarious, Dr. Barnes' case................ 248
Mercury, Medicus on .......... 260
Mercury, Dr. Ainslie's opinions on its use .................. 399
Metastasis, simple *rationale* of .. 323
Milan—the pellagra............ 99
Mollesence of the lungs, Dr. Hastings on ................ 517
Morbid anatomy and symptomatology ...................... 54
Mortification of the great toe, fatal case of ................ 480
Motor linguæ nerve, cases of paralysis of .................. 417
Moxa in phlegmatia dolens...... 250
Moxa, Mr. Wallace's work on .. 448
Moxa, physiological action of .. 449
Moxa, modes of applying it .... 451
Moxa, friction, as an adjuvant to 454
Mucous membrane of the stomach, discoloration of........ 147
Mucous membrane, morbid softening of the................ 148
Mucous membrane, morbid growths from the............ 152

N.

Neapolitan hospitals .......... 91
Nerve of the tooth, exposed; treatment of .................... 213
Nerves, divisions of ............ 428
Nerves, contusions of.......... 431
Nerves, ligature of ............ 433
Nerves, re-union of, after division 437
Nerves, inflammation of........ 439
Neuralgies of various parts...... 442
Neuroses, do they depend on inflammation?.................. 324
Nitrate of silver in epilepsy...... 241
Non-jurors .................. 270

O.

Obstructed menses, remedy for.. 303
O'Connor, Dr. on the *bische* of Trinidad .................. 470
Operations, at St. Thomas's hospital ...................... 617
Ophthalmia, Mr. Mackenzie on 173
Orthopedia .................. 258
Oxford "Traveller-ships;" their *important* results ............ 89

P.

Paracentesis thoracis, a successful case of .................. 78
Paralysis, local, cases of ........ 446
Paralysis attendant on insanity, Dr. Chalmeil on ............ 363

Paralysis, pathology of this disease 365
Paralysis, its progress and termination.......................... 370
Paralysis of organs, some curious cases of.......................... 69
Paris, Dr. his treatise on diet .. 1
Parotid gland, extirpation of.... 564
Parricide, instructive case of.... 486
Patella, fracture of the ........ 299
Patent Vituperative Company .. 270
Pathological propositions ....... 321
Perforation and softening of the stomach.......................... 240
Pericardium wanting, a case of .. 235
Pericardium, dropsy of......... 623
Periodical medical press, retrospective review of the......... 300
Periodical peritonitis, some cases of.......................... 459
Phagedena gangrenosa, case of.. 259
Phlebitis encephalica, case of .. 330
Phlegmasia dolens, Mr. Fraser's case of.......................... 464
Phlegmatia dolens, moxa in ..... 250
Phthisis cured by mercury....... 271
Phthisis pulmonalis, objections to the term.......................... 331
Physiological propositions ...... 314
Piles, new operation for......... 271
Pleurisy and abscesses after operations.......................... 521
Pleximetre, Mr. Piorry's. ....... 496
Poisoned wounds, effects of compression in.......................... 232
Polypus uteri, Mr. Stone on ..... 210
Porter, Mr. on the surgical pathology of the larynx, &c......... 375
Portio dura and fifth pair, their functions considered ......... 67
Posterior tibial artery, puncture of the.......................... 247
Post-mortem changes, experiments on .......................... 295
Post-mortem examination of Talma .......................... 465
Post-mortem changes in mucous membranes.......................... 474
Pregnancy complicated with hydatids.......................... 270
Preservation of the teeth......... 136
Principles of dental surgery, by Mr. Koecker.......................... 129
Principles of medicine, by M. Broussais .......................... 313
Prize Hospital Report, Mr. Smith's 601
*Prostration*, treatment of this state 108
*Prostration*, individual symptoms of .......................... 111
Prussic acid in catarrh and phthisis.......................... 532
Purgation, its uses and abuses .. 504
Purgation, its powers in disease.. 604
Purpura hæmorrhagica, Dr. Fairbairn's case of.......................... 61
Purpura hæmorrhagica, Drs. Hawkins and Chambers on......... 200
Puzzling point for the bench of bishops .......................... 268

Q.

Quain, Mr. his translation of Martinet's pathology.......................... 407
Quinine in intermittents......... 606
Quinine, employed in friction.... 243

R.

Rare cases in surgery, by Scarpa 221
Re-action, excessive, after prostration .......................... 113
Recto-vesical operation, case of.. 552
Remarkable case, by Mr. Muller 239
Retention of urine—odd false passage .......................... 576
Retina, phlogosis of the......... 272
Retroversio uteri, case of ...... 259
Rheumatic ophthalmia, Mr. Mackenzie on .......................... 558
Rome, hospitals and state of medicine at.......................... 93
Rostan, M. on diagnosis and prognosis .......................... 51

S.

Scalp-wounds, cases of, with remarks.......................... 191
Scarpa's rare cases in surgery.... 221
Sciatica, cured by turpentine.... 577
Scirrhus, constitutional predisposition to.......................... 552
Secale cornutum, Mr. Haslam on, 517
Sensibility, various kinds of...... 71
Silk-worm gut for ligatures .... 249
Smith, Mr. his Prize Hospital Report .......................... 601
Sore throat peculiar to children, Dr. Hamilton on ............ 223
Spasm of the bladder in lithotomy 265
Sphacelated urethra, supposed regeneration of.......................... 220
Spine, fracture of, trepanned.... 601
Spine, injuries of, at St. Thomas's, 601
Spine, case of fracture of the.... 605
Stomach; morbid affections of its follicles .......................... 153
Stomach; diseases of its cellular coats .......................... 156

Stomach; cases of enormous distention of the .............. 159
Stomach, case of chronic ulcer in the ...................... 493
Stomach, softening and perforation of.................... 240
Stone, Mr. on polypus uteri ..... 210
Stopping of carious teeth, remarks on ....................... 143
Strangulated hernia, case of .... 609
Stricture of the ileum, Dr. Moleson's case of ............... 83
Structure sliced in search of function...................... 262
Subcarbonate of iron, M. Duparque on .................. 252
Subclavian, M. Dupuytren's ligature of the................. 163
Sudden and mysterious death.... 251
Surgical report of M. M. Roux, Breschet, and Dupuytren .... 548
Surgical clinique of St. Louis.... 560
Syncope anginosa................ 574
Sympathy, Dr. Alison's observations on.................. 63

T.

Talma, post-mortem examination of ......................... 465
Teeth, effect of the general health on the ..................... 132
Tetanus, tobacco in............. 298
Tetanus, mercurial inunction in 304
Tetanus, some cases of.......... 211
Tetanus, case of, and lecture on ......................... 614
Thorn, Mr. his extract of bals. copaibæ ..................... 499
Thymus gland, Mr. Wood on disease of the................. 477
Transactions of the Edinburgh Society..................... 61
Transfusion, another successful case of ................... 575
Travels in Italy in 1820-24 by Dr. Valentin.................. 88
Travers, Mr. on constitutional irritation ...................... 101
Treatment of indigestion........ 39
Trepanning of the spine, case of 601
Trephine, question of the, considered .................... 171
Trephine, observations on its use, 263
Tubercular abscesses after operations, cases of .............. 524
Tumours of nerves ............ 441
Tyrrell, Mr., operations on the spine ...................... 601

U.

Ulcers and burns, chloruret of lime in ......................... 360
Ulcers, simple and varicose, M. Lisfranc on ................. 538

V.

Vaccination, Dr. Gregory on .... 192
Valentin, Dr. his Voyage en Italie 88
Vascular inflammation, general, cases of .................... 501
Vein, excision of a portion of, in varicose legs ............... 539
Velpeau, M. on pleurisy and abscesses after operations ...... 521
Venereal lepra after gonorrhœa, case of ................... 606
Venesection, accidents from .... 193
Vesical fistula, case of, with remarks...................... 257
Vicarious menstruation, case of.. 248
Viper-bite, M. Piorry's case of ... 468

W.

Wallace, Mr. his work on moxa.. 448
Webster, Dr. his case of cherry-stone in the bronchia ........ 228
White-mustard seed, letter on its employment ................ 512
Whytt, Dr. his anticipation of Dr. Philip...................... 303
Wound of the heart, a case of... 245
Wounds of nerves.............. 426

---

N. B. It will be seen that we have added a spare title page to each volume, for the convenience of those Subscribers who commence with the present year.

